# Modelling the probability and impact of false‐positive serology for *Borrelia burgdorferi* sensu lato: A case study

**DOI:** 10.1111/evj.13277

**Published:** 2020-06-23

**Authors:** Rosa M. A. C. Houben, Carole Meersschaert, Guy Hendrickx, Pierre‐Hugues Pitel, Hélène Amory

**Affiliations:** ^1^ Department of Equine Sciences Faculty of Veterinary Medicine Utrecht University Utrecht The Netherlands; ^2^ Laboratoires Réunis Junglinster Luxembourg; ^3^ Avia‐GIS Zoersel Belgium; ^4^ LABEO Frank Duncombe Caen France; ^5^ FARAH Faculty of Veterinary Medicine Liège University Liège Belgium

**Keywords:** horse, Lyme, Borrelia, seroprevalence, misdiagnosis, antimicrobial use

## Abstract

**Background:**

Serological screening tests for Lyme borreliosis have poor specificity, with potential for misdiagnosis and unnecessary antimicrobial treatment.

**Objectives:**

To evaluate the impact of Lyme borreliosis seroprevalence and serologic test characteristics on the probability of obtaining a false‐positive result and impact on antimicrobial use.

**Study design:**

Cross‐sectional serological survey and modelling.

**Methods:**

Sera from 303 horses in southern Belgium were analysed by enzyme‐linked immunosorbent assay (ELISA). Apparent seroprevalence was derived from serological data and a Bayesian estimate of true seroprevalence was computed. These were a starting point to model the impact of test and population characteristics on the probability of obtaining false‐positive results and consequently unnecessary treatments and complications.

**Results:**

Apparent and true seroprevalence were 22% (95% CI 18%‐27%) and 11% (credible interval with 95% probability 0.6%‐21%) respectively. We estimate that two‐thirds of positive samples are false positive in southern Belgium, with one in five of tested horses potentially misdiagnosed as infected. Around 5% of antimicrobial use in equine veterinary practice in Belgium may be attributable to treatment of a false‐positive result.

**Main limitations:**

There was uncertainty regarding the ELISA's sensitivity and specificity.

**Conclusions:**

This study highlights the importance of appreciating the poor diagnostic value of ELISA screening for Lyme borreliosis as demonstrated by this case study of seroprevalence in southern Belgium where we demonstrate that a nontrivial number of horses is estimated to receive unwarranted treatment due to poor appreciation of screening test characteristics by practitioners, contributing substantially to unnecessary use of antimicrobials.

## 1 INTRODUCTION

Lyme borreliosis is the most prevalent tick‐borne infection in the northern hemisphere, affecting both humans and animals.^1^, ^2^, ^3^ Reported seroprevalence of *B burgdorferi* among horses in Europe varies widely from 6% to 36%.^4^, ^5^, ^6^, ^7^, ^8^, ^9^, ^10^, ^11^ This variation may be not only caused by geographical differences in seroprevalence but also by the characteristics of the diagnostic tests used to assess prevalence in these studies.

In Europe, enzyme‐linked immunosorbent assay (ELISA) and immunofluorescence assay (IFA) are routinely used for serologic confirmation of exposure to *B burgdorferi* ^12^ and both tests are currently commonly used in Belgium.

Both tests are considered sensitive but not particularly specific and in both human and veterinary medicine, a two‐tier approach to serological diagnosis of exposure to *Borrelia* spp is recommended whereby a positive screening test such as ELISA is followed by a confirmatory, more specific test, usually an immunoblot^2^, ^12^, ^13^, ^14^. Divers et al^2^ have reviewed how while serological confirmation of exposure to *Borrelia* spp is straightforward, it does by no means confirm Lyme borreliosis and further investigations are required before a diagnosis of Lyme borreliosis and subsequent treatment are decided on. The recently published Consensus Statement on Lyme disease describes this as follows: “*Treatment of nonclinical, seropositive horses will result in the unnecessary treatment of many horses resulting in unnecessary expense, increased risk of adverse events and inappropriate use of antimicrobials*”.^2^ However, awareness of the pitfalls of diagnosis of Lyme borreliosis may not be widespread among veterinarians and, in the authors’ experience, horses are frequently treated with antimicrobials following a positive result on a screening test. If the screening result was actually a false positive, there are other negative consequences to consider besides unwarranted antimicrobial use and resulting potential for adverse effects associated with treatment,^15^ such as nontreatment of the actual condition.

The first aim of our study was to estimate the seroprevalence of antibodies to *B burgdorferi* in horses without clinical signs suggestive of Lyme borreliosis in southern Belgium. The second aim was to explore the impact of Lyme borreliosis test characteristics on test interpretation and to estimate the impact of poor test specificity on potential antimicrobial use.

## 2 MATERIALS AND METHODS

### 2.1 Survey population

A serological survey was carried out. The population under investigation were all horses housed in southern Belgium which according to the national horse registry (CBC) stood at 73 772 in 2014. All participating horse owners were asked to complete a questionnaire (Data S1). Horses were only included in the study if they were aged at least 12 months and had been housed at their current location for at least the preceding 12 months. Horses were excluded if their owner ticked “yes” on any of the boxes indicating that the horse had, in the preceding 12 months, presented clinical signs that are (or have been in the past) attributed to *B burgdorferi* infection in horses. The questionnaire also recorded whether horses had pasture access and whether a tick had been found on the horse in the preceding 12 months, but these data were not used in the current study. Samples were either derived from horses presented to the Liege University's equine hospital accompanying hospitalised foals, for elective surgical procedures, traumatic injury, cardiologic evaluation, simple obstructive or strangulating acute colic, or alternatively were collected in the field.

### 2.2 Serological analysis

Blood was collected via jugular venipuncture into plain serum tubes, allowed to clot, centrifuged and frozen at −80°C until analysis or, for samples collected in the field, stored first at −20°C then within 1 week transferred to −80°C until analysis. Samples were collectively transported frozen to the laboratory (LABEO Frank Duncombe) and analysed by a commercially available ELISA kit (Testkit Borrelia burgdorferi Veterinary ELISA IgG, Virotech Diagnostics).

In a prior study,^16^ we evaluated the performance of this ELISA kit using Western Blot (WB) (Borrelia veterinär plus OspA LINE, IgG Line Immunoblot, Sekisui Virotech GmbH) as a reference test, calculating sensitivity and specificity using two possible ELISA cut‐offs for seropositivity. The manufacturer's specifications for interpretation are as follows: negative (<8 Virotech Units or VE), intermediate (≥8 and <12VE) and positive (≥12VE). When all ELISA samples of ≥8VE were considered positives, ELISA sensitivity and specificity were 96 and 63% and this was the test interpretation applied to our seroprevalence estimation. For the purpose of our potential overtreatment modelling scenario, we wished to take a more conservative approach and counted only ELISA results of ≥12VE as positives. Applying that test interpretation, ELISA sensitivity and specificity were 83 and 78%.

It should be noted that for both ELISA test interpretations applied in this study, sensitivity and specificity were substantially lower than the test manufacturer's specifications of 100% and 96%.

### 2.3 Sample size

A power calculation was performed to estimate the number of horses to sample. As no prior data on seroprevalence among horses in southern Belgium were available, we opted to carry out a preliminary study on serology by IFA and found 15 positives among 67 horses with no signs suggestive of Lyme borreliosis. For a seroprevalence of approximately 22%, a sample size of at least 264 animals is required to estimate the population's seroprevalence with a 95% CI width of 10%.^17^ An equal number of horses was sampled from each of the five administrative districts of southern Belgium.

### 2.4 Data analysis

As the ELISA sensitivity and specificity from our preliminary work were markedly poorer than those indicated by the test manufacturer (96% vs 100% for sensitivity and 63% vs 96% for specificity), estimation of true seroprevalence by traditional methods^18^ was not considered appropriate due to the uncertainty around the actual test characteristics. As an alternative, we assumed that the actual sensitivity and specificity lay somewhere between our estimate and the manufacturer's. We calculated a Bayesian estimate of true seroprevalence using Markov Chain Monte Carlo simulation^19^ with prior estimates of a uniform distribution of 96%‐100% for sensitivity and of 63%‐96% for specificity.

Additionally, a χ^2^ test was applied to compare the expected *vs* actual proportion of test positive horses in the sample. All statistical analyses were performed in R (R version 3.5.2; R Core Team).^20^

### 2.5 Model scenarios

To explore the potential impact of test performance, we devised the following three scenarios:

#### 2.5.1 True seroprevalence

Given the test performance as determined in our prior work, the proportion of positive tests which is most likely a false positive was calculated for a range of seroprevalences up to the highest reported in Europe (range 0%‐36%).

#### 2.5.2 Test performance

The effect of varying test specificity and sensitivity between the manufacturer's specifications and our own estimates on the proportion of positive tests that is most likely a false positive was evaluated (sensitivity range 96%‐100% and specificity range 63%‐96%), with seroprevalence fixed at the point estimate for true seroprevalence from our seroprevalence study.

#### 2.5.3 Potential overtreatment

The impact on antimicrobial use if every horse with a positive (≥12VE) ELISA result is treated with a 21‐day course of antimicrobials is explored. The risk of serious complications following overtreatment was calculated assuming an incidence of 0.6% per course of antimicrobials administered to a horse.^15^ In addition, the potential relative contribution of overtreatment on overall antimicrobial use was calculated using local data on average daily doses per horse per year^21^ and number of tests submitted annually for Lyme borreliosis screening serology to one regional laboratory.^22^

To evaluate whether this overtreatment scenario was likely to happen at all, given the available information in veterinary scientific literature on the pitfalls of Lyme borreliosis diagnosis, we conducted a small survey by issuing a questionnaire (Data S2) to veterinarians visiting Belgian equine (BEPS) or general veterinary (Véterinexpo, AVPL) conferences in the fall of 2019.

## 3 RESULTS

Between May 2014 and April 2016, 303 samples were collected of which 155 were collected from horses presented to the Liege University's equine hospital and 148 were collected in the field. The horses included in the study were 139 mares, 127 geldings and 37 stallions, with ages ranging from 1 to 31 years (median 11 years, interquartile range 7‐17 years). The ELISA results were as follows: 235 samples were <8VE, 29 were ≥8VE and <12VE, and 39 samples were ≥12VE. Using the cut‐off of ≥8VE, apparent seroprevalence for *B burgdorferi* was 22 (95% CI 18‐27) percent.

The proportion of positive test results in our sample was lower than expected when taking into account our prior estimates of ELISA specificity and sensitivity. Had true seroprevalence been zero, assuming a test specificity of 63% we still would have expected 112 of 303 samples to be false positive. This difference was significant (χ^2^ of observed 68/303 vs expected minimum 112/303 was *P* = .001). The Bayesian estimate for true seroprevalence was 11% (credible interval with 95% probability CI 0.6%‐21%).

### 3.1 Scenarios

#### 3.1.1 Impact of true seroprevalence

**FIGURE 1:**
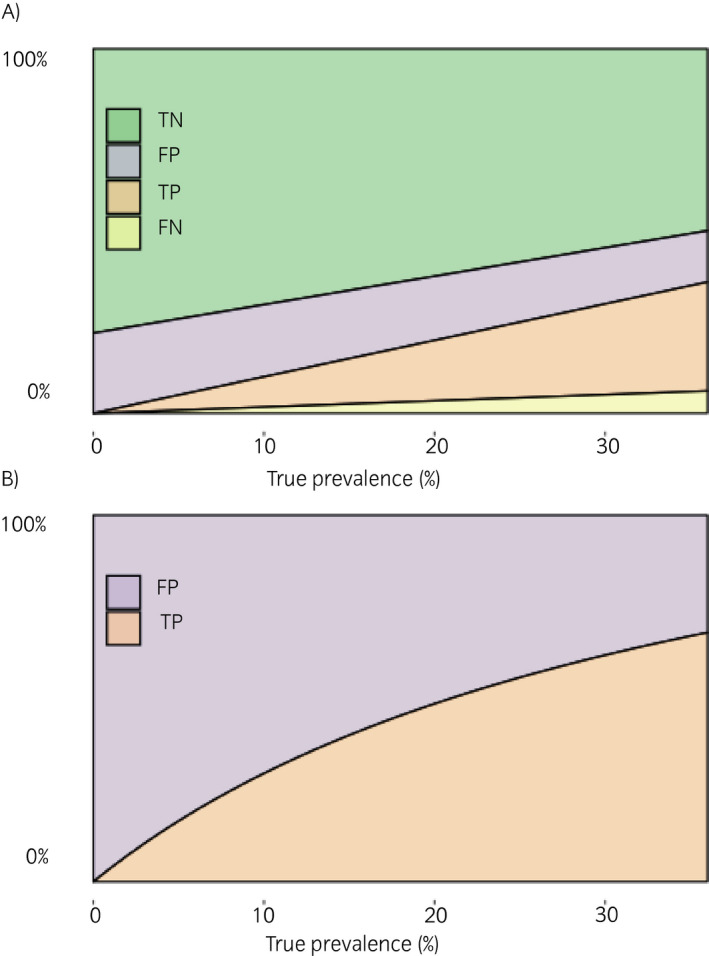
Expected distribution of true negative (TN), false positive (FP), true positive (TP) and false negative (FN) for all submitted samples (A), and of all positive test results (B) for fixed test characteristics of 83% sensitivity and 78% specificity, and varying true seroprevalence over the range of seroprevalences reported in Europe

The expected distribution of true‐negative, false‐positive, true‐positive and false‐negative results obtained when test sensitivity and specificity are 83 and 78%, and population true seroprevalence varies, is illustrated in Figure 1. When true prevalence is 11% such as in southern Belgium, then more than two‐thirds of all positive samples will be false positives. Therefore, with these test characteristics, any positive serological result in a horse residing in southern Belgium is more likely to be a false positive than a true positive. Predictably, this ratio improves as true seroprevalence increases. However, it is important to realise that even in populations with the highest reported seroprevalence in Europe, almost one in three (32%) of positive results is likely a false positive.

#### 3.1.2 Impact of test performance

**FIGURE 2:**
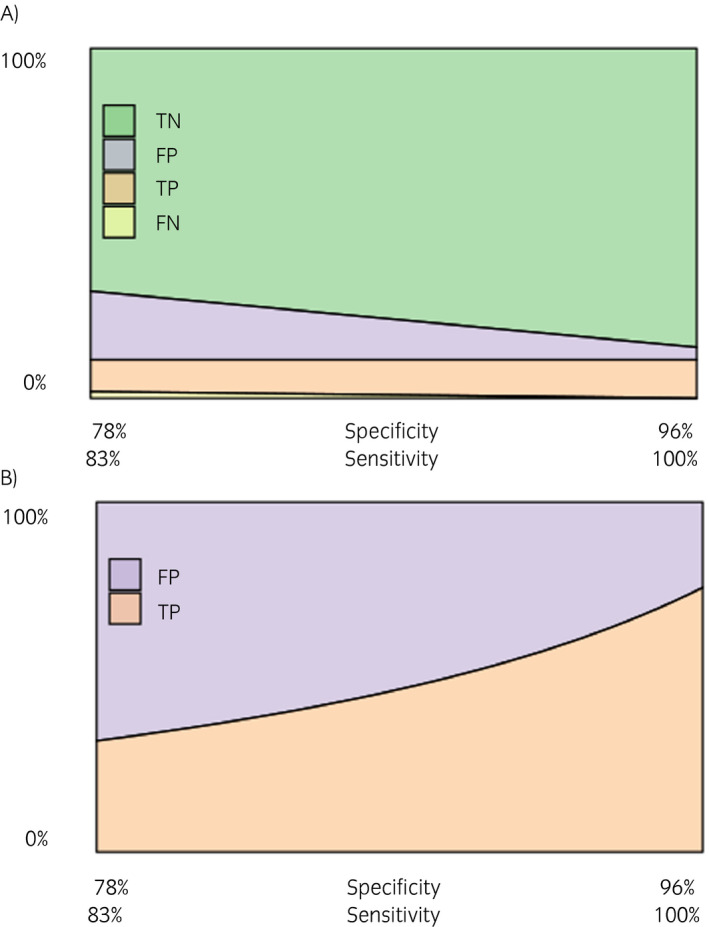
Expected percentages of (A) true‐negative (TN), false‐positive (FP), true‐positive (TP) and false‐negative (FN) samples overall and of all positive test results (B), for a fixed true prevalence of 11% and varying test sensitivity and specificity

The expected distribution of test true‐negative, false‐positive, true‐positive and false‐negative results obtained by fixing true prevalence at 11% and concurrently increasing sensitivity and specificity from 83% to 100% and from 78% to 96% is illustrated in Figure 2. The actual performance of the ELISA kit remains unknown, as outlined previously. The steeper curve towards the right of Figure 2B highlights how especially among higher values, a small deviation from the manufacturer's specifications of specificity has a relatively large impact on the probability of a positive test being a false positive.

#### 3.1.3 Potential overtreatment

We assume a true seroprevalence of 11%, a test sensitivity and specificity of 83 and 78% (which corresponds to the more conservative interpretation of considering an “intermediate” ELISA result a negative result), an incidence of antimicrobial associated diarrhoea of 0.6% with a case fatality of 19% per course of antibiotics administered to a horse.^15^ For every 1000 tests submitted, 91/1000 horses will be correctly identified as seropositive and 196/1000 (68% of all horses that have tested positive) will produce a false‐positive result and be at risk of unwarranted antimicrobial therapy. In this scenario, one case of antimicrobial‐associated diarrhoea due to unwarranted antimicrobial use is expected for every 850 horses screened for Lyme borreliosis and one fatal outcome of antimicrobial‐associated diarrhoea for every 4475.

In an earlier study,^22^ records from a commercial regional Belgian laboratory indicated that, in 2014, 507 equine serum samples were submitted for Lyme borreliosis screening. Of these 507 tests, 164 returned a positive result. This number of positives is only slightly higher than we would have expected if the samples had been taken from apparently healthy horses such as our study population, in which case we would have expected 146 seropositive horses and the difference was nonsignificant (χ^2^ *P* = .3). Of these seropositive horses, we estimate that 111 may have been false positives and if treated, these horses would have received 2331 (21 * 111) daily doses of antimicrobials. Assuming that the 73 772 horses housed in southern Belgium in 2014 received, on average, a similar amount of antimicrobials per horse as those in the Netherlands (ie total use = 73 772 * 0.562 = 41 460 doses per year), then 5.6% (2331/41460 * 100) of all daily doses of antimicrobials administered to horses in Belgium that year could have been attributable to overtreatment following a false‐positive Lyme borreliosis screening test result.

Out of 100 responses from Belgian veterinarians to our survey presenting a case of a horse with nonspecific complaints and a positive borreliosis screening tests, 60 respondents chose to treat the horse with antimicrobials.

## 4 DISCUSSION

In the serosurvey part of our study, apparent and true seroprevalence were 22% (95% CI 18%‐27%) and 11% (credible interval with 95% probability 0.6%‐21%), which falls within the range of reports of 6%‐36% in other regions in Europe.^4^, ^5^, ^6^, ^7^, ^8^, ^9^, ^10^, ^11^ It should be noted that in many of these studies, performance characteristics of the test used to establish serological status are not discussed and most seem to report apparent seroprevalence.

The much lower than expected number of ELISA positives among the horses we sampled was surprising and may indicate a difference in test performance in the healthy horses we sampled in this study, compared to the group of horses in which test characteristics were evaluated, suggesting that specificity of ELISA is poorer if applied to horses suspected of Lyme borreliosis than if applied to horses without signs suggestive of Lyme borreliosis. For this earlier study in which sensitivity and specificity for the ELISA kit were estimated, samples were derived from both healthy horses as well as horses with a clinical suspicion of Lyme borreliosis.^22^, ^25^ The age of the horses in this comparison study was not recorded, however, and we cannot exclude the possibility that a lower mean age of participants in our seroprevalence study than the participants in the comparison study was a contributing cause for the lower than expected number of seropositives. This would be due to older horses being more likely to test positive for borreliosis, as was also the case in our seroprevalence study (Data S3).

As our findings illustrate, serologic tests may perform worse than claimed in the population they are actually applied to. Such was the case for the ELISA kit used in our study, but the same may apply to other screening tests such as IFA, which generally has test characteristics similar to our ELISA.^22^, ^25^ Another screening test which is available in most of Europe is the C6 Snap Test, an antigen‐specific patient‐side ELISA kit. The C6 Snap Test is reported to have a sensitivity of 93% and a specificity of 96% in clinical settings in North America.^23^ So far, reports over its performance and its specificity in particular have varied in studies on North American horses.^23^, ^24^ It is likely that at least some of this variation is due to differences in the population they are evaluated in, as perfect specificity was found in a population of pathogen‐free ponies,^24^ whereas in a patient population specificity was good at 96% but by no means perfect.^23^ Cross‐reactivity with other (spirochete) pathogens could be a possible explanation for the discordance between these estimates of specificity.^23^ The difference in relative importance of the different *Borrelia* spp (sensu stricto vs sensu lato) in North America vs Europe may also affect test performance characteristics of the C6 Snap test if it is applied in Europe. Butler et al^25^ evaluated the C6 Snap Test by applying the test alongside ELISA and IFAT to horses from which ticks had recently been removed. Although the population this comparison was applied to differed from our study's sample population as we did not select for tick infestation, the results presented by Butler *et al*. do not suggest that the C6 Snap Test would have performed much better than the ELISA had we applied it to our study population.

Our modelling estimates indicate that among healthy horses residing in southern Belgium, around one in five ELISA tests will return a positive result but two‐thirds of these will be false positives, due to a combination of poor test specificity and a low true seroprevalence. If ELISA is solely relied upon for a diagnosis of Lyme borreliosis, this has important implications on antimicrobial use in equine veterinary practice. In our (arguably pessimistic) scenario of treatment of every horse with a positive test result, around 5% of antibiotics used on horses annually could be attributable to treatment following inappropriate interpretation of *Borrelia* screening serology. For this estimate, we applied per horse at risk of treatment per year sales data from neighbouring country the Netherlands, as similar data were, unfortunately, not available for Belgium. Data from the European Medicines Agency^26^ indicate that antimicrobial use patterns differ between countries, and such appears to be the case for Belgium and the Netherlands (eg 29 mg of tetracyclines was used annually per kg of food‐producing animal including horses in Belgium, vs 20 mg in the Netherlands). However, we believed that the horse‐specific and per‐daily‐dose sales data available from the Netherlands were the most appropriate data to use for our estimation of the contribution of false‐positive serology on unwarranted antimicrobial use.

With regards to our overtreatment scenario, in reality it is of course unlikely that every horse that tests positive on a screening test receives antimicrobial treatment without further investigations. The results from our survey among veterinarians, however, suggest that the scenario is not that far from reality. Sixty per cent of veterinarians who completed the survey questionnaire chose to prescribe antimicrobials to a fictional case with nonspecific complaints of poor performance and a positive *Borrelia* titre in a horse. No effort was made for the veterinarians’ survey to obtain a truly cross‐sectional sample; respondents self‐selected first by attending a veterinary conference and then by also volunteering to participate in our survey. Antimicrobial prescribing may be different in veterinarians who do neither of those, and it is plausible that those who were not surveyed, may be even more likely to prescribe antimicrobials under a similar scenario.

Our 5% estimate may have overestimated the proportion of veterinarians who would prescribe antimicrobials, but it certainly underestimated the number of serologic tests that were carried out, as *Borrelia* serology is offered by other regional laboratories besides that from which submission data were available for our study. All in all, considering the above, it is likely that a nontrivial percentage of total equine antimicrobial use per annum has followed a false‐positive *Borrelia* serology. This indicates that addressing the understanding of the relevance of test results for Borreliosis or improving veterinarians’ resistance against horse owners demanding treatment after positive serology could be a target for regulators wishing to reduce antimicrobial use. The impact of unwarranted treatment of suspected Lyme borreliosis is relatively high, which is in part due to the prolonged course of antimicrobials that is usually prescribed.

Antimicrobial‐associated diarrhoea arising as a complication following unwarranted antimicrobial treatment was estimated to affect only a very small number of tested horses, mostly because incidence of antimicrobial‐associated diarrhoea is low in general. However, it is a complication which when it occurs is often costly to treat and has a fatal outcome in almost one in five cases.^15^

Overall, a major limitation in most of our estimates is the absence of a gold standard for serological confirmation of exposure to *B burgdorferi*. We used WB as a reference test but it is possible that some samples were misclassified by this WB.^27^ Alternative serological tests are available^2^ and may be more accurate than WB and therefore a more appropriate reference standard,^27^ however, no other tests were readily available at any of the regional laboratories at the time of this study. And, as pointed out previously, whether these tests, which are mostly aimed validated for North American horses, would actually have outperformed the WB is not sure.

Another limitation is in the construction of the owner questionnaire based on which horses were accepted or rejected for the study. As the questionnaire was aimed at horse owners of various backgrounds, assessment for signs of the accepted syndromes Lyme borreliosis of neuroborreliosis, uveitis and pseudolymphoma was attempted in a circumvent way; also, we decided to include signs suggestive of polysynovitis, although it is debatable whether this is truly a signs of joint *Borrelia* infection.^2^ Weight loss as a proxy for muscle atrophy, a change in demeanour, lamenesses and stiffness owner‐diagnosed as laminitis were all used as exclusion criteria for potential neuroborreliosis. In retrospect, excluding mares that had aborted in the preceding 12 months was unnecessary. We also insufficiently inquired about signs suggestive of cutaneous pseudolymphoma in the questionnaire. However, given the rarity of cutaneous lymphoma, the authors believe that the aforementioned limitations of the owner questionnaire do not substantially affect the validity of our findings.

### 4.1 Conclusion

Apparent seroprevalence for *Borrelia* sensu lato in horses without clinical signs suggestive of Lyme borreliosis in southern Belgium was at 22%, similar to the range of seroprevalences reported in other European countries and northern Belgium. True seroprevalence was estimated at 11%. The positive predictive value of ELISA can be expected to be poor in this population and around two‐thirds of positive test results in horses in southern Belgium are likely to be false positives. Specificity of ELISA may be poorer in horses suspected of lyme borreliosis than in healthy horses. These limitations should be taken into account when interpreting test results. We have demonstrated that around 5% of antimicrobials used in equine veterinary practice in southern Belgium could be attributable to treatment following incorrect interpretation of a positive screening test result and have thus highlighted an opportunity for antimicrobial use reduction.

This study did not aim to quantify the impact of incorrectly considering a truly positive *Borrelia* serology, and therefore evidence of infection, as sufficient proof of Lyme borreliosis. The authors reiterate the advice to follow Consensus recommendations^2^ for reaching a diagnosis of Lyme borreliosis before deciding on any treatment.

## 5 DATA ACCESSIBILITY STATEMENT

The data that support the findings of this study are available from the corresponding author upon reasonable request.

## CONFLICT OF INTERESTS

C. Meersschaert and P. Pitel are employed by commercial laboratories which offer *Borrelia* diagnostics. Neither laboratory had influence on the content or the submission of this manuscript.

## AUTHOR CONTRIBUTIONS

R. Houben, H. Amory, G. Hendrickx and C. Meersschaert designed the study. H. Amory and C. Meersschaert collected and collated the data. Sample analysis was by C. Meersschaert and P. Pitel. R. Houben analysed the data and prepared the manuscript with input from C. Meersschaert, H. Amory, G. Henrickx and P. Pitel.

## ETHICAL ANIMAL RESEARCH

The study protocol was approved by the Liege University's Ethical committee (reference 2015/258).

## OWNER INFORMED CONSENT

All owners consented to participation in the study.

## Supporting information

Data S1

Data S2

Data S3
