# Supplementary material for: Modelling the probability and impact of false‐positive serology for Borrelia burgdorferi sensu lato: A case study
Source: Equine Vet J. 2020 Jun 23;53(1):71–7. doi: 10.1111/evj.13277 (PMC7818418; doi:10.1111/evj.13277)
Supplement: Supplementary file 1 — Data S1 [file EVJ-53-71-s001.pdf]

Supplementary Item 1: Owner questionnaire

Lyme seroprevalence study : Animal history

Sampling date : ……………………………………………………

Veterinarian : ……………………………………………………………………

Horse :

- Name : ……………………………………………………………………………
- Sex : ☐ stallion ☐ gelding ☐ mare
- Breed : ………………………………………………………………………………
- Age : …………………

Owner :

- Name : ……………………………………………………………………………
- Address : ………

………

PLEASE NOTE : THE HORSE MAY NOT HAVE LEFT BELGIUM IN THE PREVIOUS 12 MONTHS TO BE INCLUDED IN THE STUDY

Has the horse or pony had pasture access in the last 12 months ?

☐ Yes ☐ No

If yes, where was this pasture located ?

………

………

………

In the last 12 months, has the horse or pony presented any of the following :

- Fever or dullness of unknown cause? ☐ Yes ☐ No
- Swollen joints or lameness of unknown cause? ☐ Yes ☐ No
- Episode of laminitis of unknown cause? ☐ Yes ☐ No
- Weightloss of unknown cause? ☐ Yes ☐ No
- Eye problems? ☐ Yes ☐ No
  - If yes, please describe :
- Have ticks been found on the animal? ☐ Yes ☐ No
- If the animal is a mare, has she aborted in the last 12 months? ☐ Yes ☐ No

Has a serologic test for borreliosis been carried out in the past 12 months ? ☐ Yes ☐ No

If yes, what was the result ?

Any other remarks :
