# Supplementary material for: Modelling the probability and impact of false‐positive serology for Borrelia burgdorferi sensu lato: A case study
Source: Equine Vet J. 2020 Jun 23;53(1):71–7. doi: 10.1111/evj.13277 (PMC7818418; doi:10.1111/evj.13277)
Supplement: Supplementary file 2 — Data S2 [file EVJ-53-71-s002.pdf]

Equine Veterinary Journal BEVA

Supplementary Item 2: Referring veterinarian questionnaire.

General information

Where do you practice (country and province)?

Whis animal species do you treat (multiple answers possible) ?

Companion animals / small animals

Horses

Ruminants

Porcine

Other, namely:

What year did you qualify as a veterinarian?

Do you practice o alone o in a group practice

Case 1

You are consulted about about a 6 year old warmblood mare, used for recreational riding. She has presented with a cough and serous nasal discharge since of about 12 hours duration. The mare is housed on a yard with a total of 15 horses, 5 of which have presented a cough and nasal discharge in the preceding week.

What is your advice to the owner (multiple answers possible)?

Treat the horse with PO TMS

Treat the horse with IM penicillin

Treat the horse with IV gentamicin

Treat the horse with PO doxycycline

Treat the horse with IM ceftiofur

Treat the horse with NSAIDs

Perform additional diagnostics, namely:

Other, namely:

Case 2

You are consulted about a 10 year old female sport horse which has pasture access for about 5 hours each day. The owner's complaint is that since the past 5 weeks, the mare has been slightly dull in demeanor and her performance in practice and competition has diminished slightly. There are no abnormal findings on general clinical exam. You elect to send in bloodwork and receive the following results: a neutrophilic leukocytosis and a positive (1:1024 IFA) titer for *Borrelia*.

What is your advice to the owner (multiple answers possible)?

Treat the horse with PO TMS

Treat the horse with IM penicillin

Treat the horse with IV gentamicin

Treat the horse with PO doxycycline

Treat the horse with IM ceftiofur

Treat the horse with NSAIDs

Perform additional diagnostics, namely:

Other, namely:
