# Supplementary material for: Modelling the probability and impact of false‐positive serology for Borrelia burgdorferi sensu lato: A case study
Source: Equine Vet J. 2020 Jun 23;53(1):71–7. doi: 10.1111/evj.13277 (PMC7818418; doi:10.1111/evj.13277)
Supplement: Supplementary file 3 — Data S3 [file EVJ-53-71-s003.pdf]

Equine Veterinary Journal BEVA

Supplementary Item 3: Age group and proportion infected graph.

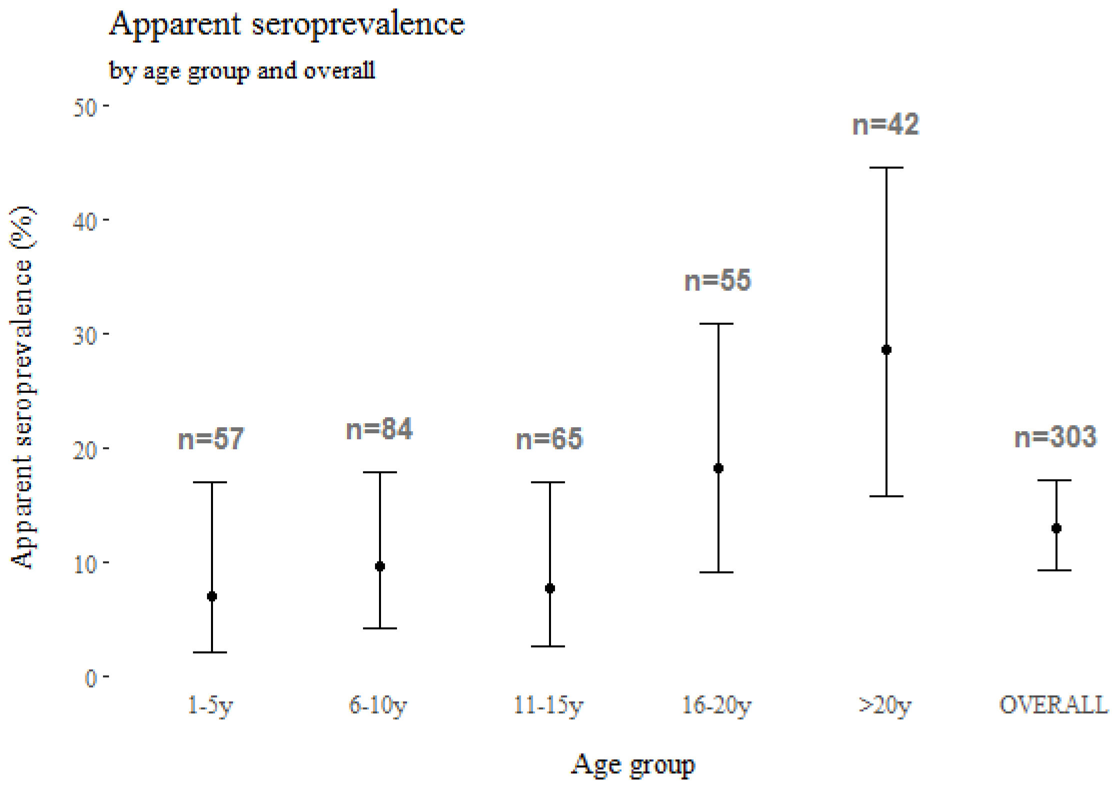

Formula call:

```
> glm(ELpos~age + gender + province, data = ldta, family = binomial)
```

Age is only significant explanatory variable for positive test result ($p < 0.001$)

Odds Ratio for each increasing year of age 1.10 (95% Confidence Interval 1.04-1.16)

For horses ≥15 years of age *vs* <15 years: Odds Ratio 2.6 ($p = 0.007$, 95% Confidence Interval 1.3-5.6)
